# Proximal Hyperdense Middle Cerebral Artery Sign Predicts Poor Response to Thrombolysis

**DOI:** 10.1371/journal.pone.0096123

**Published:** 2014-05-07

**Authors:** Qi Li, Stephen Davis, Peter Mitchell, Richard Dowling, Bernard Yan

**Affiliations:** 1 Department of Neurology, The First Affiliated Hospital of Chongqing Medical University, Chongqing, China; 2 Department of Neurology, Royal Melbourne Hospital, The University of Melbourne, Melbourne, Australia; 3 Department of Radiology, Royal Melbourne Hospital, The University of Melbourne, Melbourne, Australia; INSERM U894, Centre de Psychiatrie et Neurosciences, Hopital Sainte-Anne and Université Paris 5, France

## Abstract

The aim of our study was to compare the rapid neurological improvement after intravenous recombinant tissue-type plasminogen activator (rtPA) in patients with proximal hyperdense middle cerebral artery sign (p-HMCAS) to those without the sign and those with the distal hyperdense middle cerebral artery sign (d-HMCAS). Admission and 24 hour non-contrast CT scans of 120 patients with middle cerebral artery (MCA) territory stroke who were treated with intravenous rtPA were assessed for the presence of p-HMCAS and d-HMCAS. The sign was classified according to the site of occlusion. Rapid neurological improvement was defined as ≥50% improvement in the NIHSS score at 24 hours after thrombolysis. Rapid neurological recovery after thrombolysis was assessed and compared between the subgroups. Rapid neurological recovery was less common in the pooled group of patients with either p-HMCAS or d-HMCAS than those without the sign (p<0.01). Patients with p-HMCAS were less likely to have rapid neurological recovery than those with d-HMCAS (p<0.01). However, there was no difference in early neurological recovery between patients with d-HMCAS and those without any hyperdense sign. Our study showed that poor neurological recovery post rtPA was confined to p-HMCAS and not to d-HMCAS, indicating that these signs have quite different prognostic significance.

## Introduction

The hyperdense artery sign on admission non-contrast CT is a well established early marker of thromboembolic arterial occlusion [1], . The hyperdense artery sign has been reported in 17% to 50% of patients with middle cerebral artery (MCA) territory stroke and is associated with more severe neurological deficits, larger territory of infarction and worse functional outcome [3], [4]. Hyperdense artery sign can be categorized as proximal hyperdense middle cerebral artery sign (p-HMCAS) and distal hyperdense middle cerebral artery sign (d-HMCAS) on non-contrast CT according to the site of occlusion [4], [5].

It is well-established that thrombolytic treatment within 4.5 hours saves ischemic tissue at risk and is associated with improved neurological outcomes in patients with acute ischemic stroke [6]. In previous studies, rapid neurological improvement within 24 hours after treatment with intravenous recombinant tissue plasminogen activator (rtPA) has been reported in MCA territory stroke patients [7], [8]. Rapid neurological improvement, which was defined as ≥50% improvement in the National Institute of Health Stroke Scale (NIHSS) score within 24 hours of thrombolysis, was observed in a significant proportion of patients treated with intravenous rtPA and is associated with good clinical outcome [9]. However, the efficacy of thrombolysis in patients with hyperdense artery signs remains unclear.

In previous studies, the hyperdense artery sign was not differentiated into proximal and distal involvement. A small study suggested that patients with hyperdense artery sign might benefit from intravenous rtPA as compared with placebo [10]. However, patients with p-HMCAS and d-HMCAS were pooled in the study. Recent studies suggest that p-HMCAS and d-HMCAS carried different prognostic implications [11]. There are limited data regarding rapid neurological improvement after treatment with rtPA in patients with the hyperdense artery sign and no previous study has analysed rapid neurological recovery with reference to the location of the HMCAS.

The aim of our study was to compare the rapid neurological improvement after intravenous rtPA treatment in patients with p-HMCAS, d-HMCAS and those without the sign. We hypothesized that patients with p-HMCAS have poorer rapid neurological recovery after rtPA than those with d-HMCAS.

## Methods

This study was approved by the Ethics Committee of the Royal Melbourne Hospital and informed consent was obtained. Consecutive patients with MCA territory stroke who were treated with intravenous rtPA at the Royal Melbourne Hospital between October 2007 and October 2010 were included. All patients were treated with intravenous rtPA at 0.9 mg/kg within 3 hours of the onset of symptom and then 4.5 hours after approval of the extended time window.

The patient data were recorded in a specific stroke registry which includes patient demographics, vascular risk factors, laboratory and relevant outcome variables. Baseline non-contrast CT scans were performed within 4.5 hours after onset of symptoms. Follow-up non-contrast CT scans were performed 24 hours after the intravenous rtPA treatment. All CT scans were performed by using a Siemens Sensation scanner (Siemens Sensation 64, Erlangen, Germany), covering the whole brain. The technical parameters of the CT acquisition were as follows: 100 kv, 200 mAs, pitch 1.25, 0.5 sec rotation time, slice thickness 0.75 mm, and slice spacing 0.4 mm.

The All CT scans were independently reviewed by two independent experienced neurologists who were blinded to the clinical information. In case of disagreement, they discussed until a consensus was reached. The admission non-contrast CT scans were assessed for the presence of p-HMCAS, d-HMCAS and early ischemic changes.

**Figure 1:**
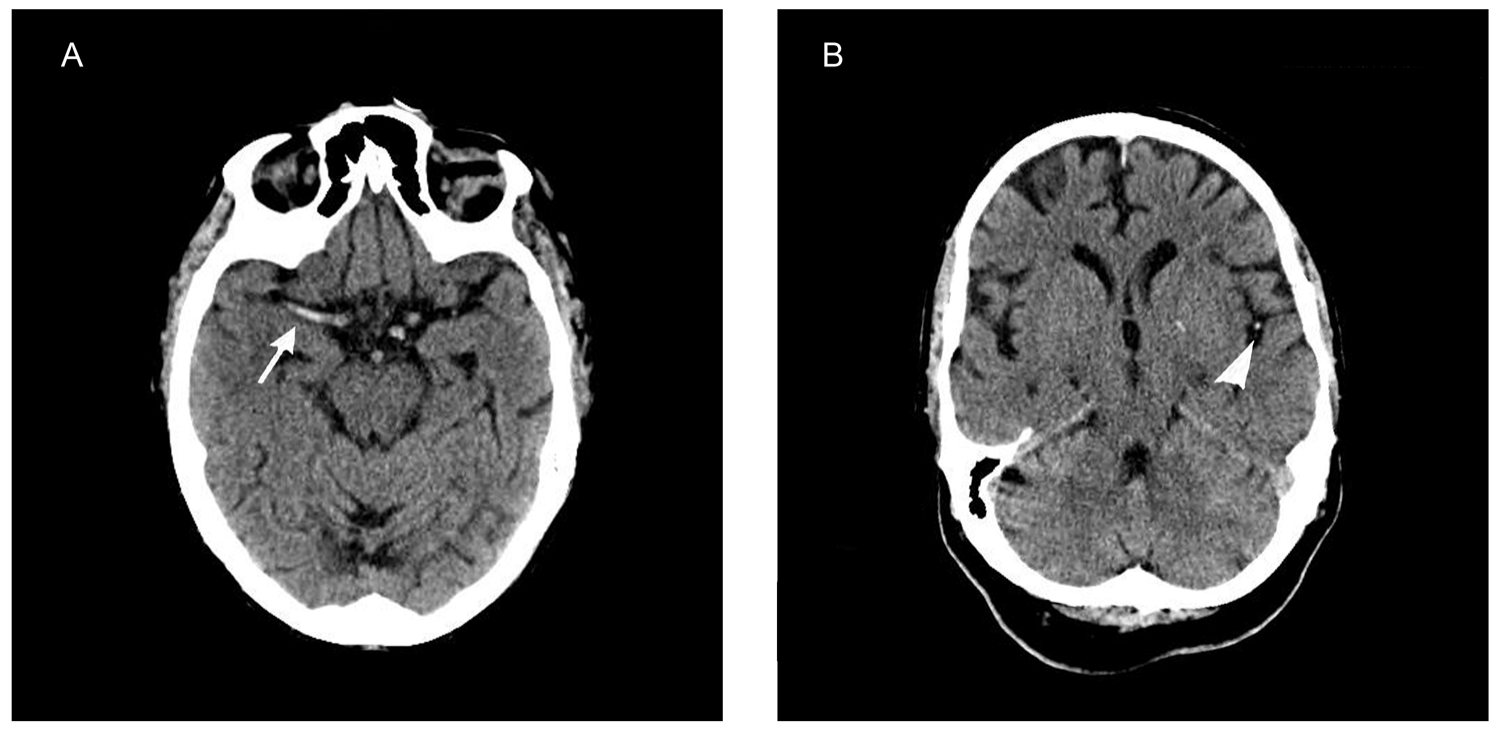
Hyperdense artery sign on noncontrast CT. (A) Transverse CT scan showing a proximal hyperdense artery sign (arrow) in the M1 segment of MCA. (B) Noncontrast CT showing a distal hyperdense artery sign in the sylvian fissure (arrowhead), which was also referred to as the MCA dot sign.

The p-HMCAS was defined as hyperattenuating signals along the course of the middle cerebral artery M1 segment (Figure 1A). The d-HMCAS, which has been termed the MCA dot sign [4], was defined as hyperattenuating dot sign in the Sylvian fissure (Figure 1B). Patients with hyperattenuating signs were classified as p-HMCAS or d-HMCAS. The follow-up CT scans were compared with the baseline non-contrast CT for the persistence of hyperattenuating signs and ischemic infarcts.

The severity of stroke was assessed by using the National Institute of Health Stroke Scale (NIHSS) for each patient on admission and 24 hours after thrombolysis. The neurological improvement was estimated by the difference between admission and the 24 hour NIHSS scores. Early neurological improvement was defined as NIHSS score improvement ≥4 points at 24 hour follow-up. Dramatic neurological improvement was defined as NIHSS score improvement ≥8 points at 24 hour follow-up. Rapid neurological improvement was defined as ≥50% improvement in the NIHSS score at 24 hours after thrombolysis.

Demographic data, vascular risk factors, results of CT scans and neurological assessment were compared between patients with hyperdense artery sign and those without the sign. The chi square test was used to assess categorical variables. For continuous variables, the dependent Student's t-test was used. The possible false discovery during multiple comparisons was controlled by Benjamini and Hochberg method. Interobserver agreement for determining hyperdense artery sign was determined by calculating kappa values, with kappa value  = 0.81–1.00 defined as excellent agreement; 0.61–0.80, good agreement; 0.41–0.60, moderate agreement; 0.21–0.40, fair agreement; 0.01–0.20, slight agreement; and 0, poor agreement.

All statistical analysis was done by using the statistical product and service solutions (SPSS) 16.0 package, with assistance of a medical statistician. P value less than 0.05 was considered statistically significant.

## Results

Of the 148 patients with middle cerebral artery territory stroke who were treated with intravenous rtPA between Oct 2007 and Oct 2010, 120 patients were eligible for the final analysis. The remaining 28 patients were excluded from our study due to incomplete data at follow-up. The study population consisted of 70 men and 50 women (mean age, 73.4 years; age range, 26–94 years). For all patients, the median pre-treatment NIHSS score was 13 (IQR, 8–18). The median time to intravenous rtPA thrombolysis was 155 minutes (IQR, 121–189).

Results of baseline data and univariate comparison of early neurological improvement after thrombolysis between patients with hyperdense artery sign and those without the sign are shown in Table 1. Patient age, gender, vascular risk factors and time to treatment did not differ between patients with hyperdense artery sign and those without the sign. Patients with hyperdense artery signs have more severe neurological deficit on admission than those without the sign (p<0.05). Early ischemic changes on initial CT scans are more common in patients with hyperdense artery signs (p<0.01). Early neurological improvement by ≥4 NIHSS points was observed in 29.7% of patients with hyperdense artery sign as compared to 48.2% of patients without the sign. Dramatic neurological improvement, which was defined as NIHSS score improvement ≥8 points at 24 hours after thrombolysis, was less common in patients with hyperdense artery sign than those without the sign (p<0.05). Rapid neurological improvement, which as defined as ≥50% improvement in the NIHSS score within 24 hours of thrombolysis, was observed in 18.9% of patients with hyperdense artery signs as compared to 44.6% of patients without the sign. Patients with hyperdense artery signs are less likely to have rapid neurological recovery than those without the sign (p<0.01).

**Table 1:** Baseline data and response to rt-PA between patients with hyperdense artery sign and those without the sign.

	Hyperdense sign	No Hyperdense	P Value
No of patients	37	83	
**Demographics**			
Sex (Male)	19 (51.3%)	51 (61.5%)	0.322
Age (Mean, SD)	72(14.4)	74 (11.5)	0.348
Age (median, IQR)	74 (65–82)	75 (67–83)	0.481
**Vascular Risk Factors**			
Hypertension	29 (78.4%)	65 (78.3%)	1
Diabetes	14 (37.8%)	27 (32.5%)	0.685
IHD	10 (27.1%)	24 (28.9%)	1
AF	10 (27.1%)	30 (36.1%)	0.462
Smoking	10 (27.1%)	22 (26.5%)	1
Hypercholesterolemia	19 (51.4%)	47 (56.6%)	0.713
Previous stroke	3 (8.1%)	15 (18.1%)	0.296
Previous TIA	1 (2.7%)	9 (10.8%)	0.187
**Neuroradiological Features**			
Early Ischemic Signs	28 (75.7%)	29 (34.9%)	<0.0001
**Neurological Status**			
Admission NIHSS (median, IQR)	16 (10–21)	11(7–17)	0.023
24 hour NIHSS (median, IQR)	15 (6–21)	6 (2–13)	<0.0001
NIHSS improvement (median, IQR)	0 (0–4)	3 (2–8)	0.0001
NIHSS Recover ≥4	11 (29.7%)	40 (48.2%)	0.081
NIHSS Recover≥8	4 (10.8%)	25 (30.1%)	0.023
NIHSS Recover≥50%	7 (18.9%)	37 (44.6%)	0.0012
**Thrombolysis**			
Time to rt-PA (median, IQR)	140 (107–182)	164 (130–190)	0.123
Symptomatic hemorrhage	1 (3.1%)	5 (6%)	0.685

NIHSS indicates National Institutes of Health Stroke Scale; IQR indicates interquartile range.

Of the 120 patients treated with rtPA, hyperdense artery sign was seen in 37 (30.8%) patients. P-HMCAS was seen in 21 (17.5%) of 120 patients, whereas 16 (13.3%) patients had d-HMCAS on the initial CT scan. The p-HMCAS and d-HMCAS coexisted in 6 patients. The hyperdense artery sign disappeared in 7 of 21 patients with p-HMCAS. The MCA dot sign disappeared in 12 of 16 patients with d-HMCAS. The interobserver agreement for hyperdense artery sign was excellent (kappa value = 0.83)

Patients with p-HMCAS were compared with d-HMCAS (Table 2). Patient age, gender, vascular risk factors and time to treatment did not differ between the two groups. The p-HMCAS is associated with more severe initial neurological deficit (p<0.0001) and worse NIHSS score at 24 hours (p<0.0001). Disappearance of hyperdense artery sign is more common in patients with d-HMCAS than those with p-HMCAS (p<0.05). Patients with p-HMCAS are less likely to have rapid neurological recovery than those with d-HMCAS (p<0.01). The p-HMCAS on baseline CT scan was significantly associated with poor early neurological recovery on univariate analysis (p<0.01). Univariate analysis did not demonstrate any association of d-HMCAS with rapid neurological recovery (OR = 1.042, p = 0.941).

**Table 2:** Baseline data and response to rt-PA between patients with proximal hyperdense artery sign and distal hyperdense artery sign.

	p-HMCAS	d-HMCAS	P Value
No of patients	21	16	
**Demographics**			
Sex (Male)	9 (42.9%)	10 (62.5%)	0.325
Age (median, IQR)	73 (69–84)	74 (62–76)	0.770
**Vascular Risk Factors**			
Hypertension	15 (71.4%)	14 (87.5%)	0.436
Diabetes	5 (23.8%)	9 (52.6%)	0.091
IHD	5 (23.8%)	5(31.2%)	0.785
AF	6 (28.6%)	4 (25%)	1
Smoking	3 (14.3%)	7 (43.8%)	0.085
Hypercholesterolemia	8 (38.1%)	11 (68.8%)	0.099
Previous stroke	1 (4.8%)	2 (12.5%)	0.576
Previous TIA	0 (0%)	1 (6.2%)	0.443
**Neuroradiological Features**			
Early Ischemic Signs	18 (85.7%)	10 (62.5%)	0.143
Disappearance of hyperdense artery sign	7(33.3%)	12 (75%)	0.028
**Neurological Status**			
Admission NIHSS (median, IQR)	19 (15–21.5)	10.5(7–15.5)	<0.0001
24 hour NIHSS (median, IQR)	19 (14.5–21)	6 (2.25–10)	<0.0001
Early NIHSS improvement (median, IQR)	0 (0–2)	3.5 (0–6)	0.035
NIHSS Recover ≥4	3 (14.3%)	8 (50%)	0.038
NIHSS Recover≥8	1 (4.8%)	3 (18.8%)	0.312
NIHSS Recover ≥50%	1 (4.8%)	6 (37.5%)	0.032
**Thrombolysis**			
Time to rt-PA (median, IQR)	164(107.5–193)	133.5 (102.5–162.75)	0.255

NIHSS indicates National Institutes of Health Stroke Scale; IQR indicates interquartile range.

Patients with d-HMCAS were compared with those without any hyperdense artery signs (Table 3). The patient demographics and vascular risk factors did not differ between the two groups. Early ischemic signs are approximately twice as common in patients with d-HMCAS. The admission and 24 hour NIHSS score were virtually the same between patients with d-HMCAS and those without any hyperdense sign. The rate of rapid neurological improvement was similar in patients with d-HMCAS and those with no hyperdense sign.

**Table 3:** Baseline data and response to rt-PA between patients with d-HMCAS and patients without any hyperdense sign.

	Solitary d-HMCAS	No Hyperdense	P Value
No of patients	16	83	
**Demographics**			
Sex (Male)	10 (62.5%)	51 (61.5%)	1
Age (median, IQR)	74 (62–76)	75 (67–83)	0.332
**Vascular Risk Factors**			
Hypertension	14 (87.5%)	65 (78.3%)	0.523
Diabetes	9 (52.6%)	27 (32.5%)	0.098
IHD	5(31.2%)	24 (28.9%)	1
AF	4 (25%)	30 (36.1%)	0.573
Smoking	7 (43.8%)	22 (26.5%)	0.085
Hypercholesterolemia	11 (68.8%)	47 (56.6%)	0.271
Previous stroke	2 (12.5%)	15 (18.1%)	0.746
Previous TIA	1 (6.2%)	9 (10.8%)	1
**Neuroradiological Features**			
Early Ischemic Signs	10 (62.5%)	29 (34.9%)	0.059
**Neurological Status**			
Admission NIHSS (median, IQR)	10.5(7–15.5)	11(7–17)	0.653
24 hour NIHSS (median, IQR)	6 (2.25–10)	6 (2–13)	0.963
NIHSS improvement (median, IQR)	3.5 (0–6)	3 (2–8)	0.316
NIHSS Recover ≥4	8 (50%)	40 (48.2%)	1
NIHSS Recover≥8	3 (18.8%)	25 (30.1%)	0.556
NIHSS Recover≥50%	6 (37.5%)	37 (44.6%)	0.792
**Thrombolysis**			
Time to rt-PA (median, IQR)	133.5 (102.5–162.75)	164 (130–190)	0.082

NIHSS indicates National Institutes of Health Stroke Scale; IQR indicates interquartile range.

## Discussion

In previous reports, the hyperdense artery sign has been reported in 17% to 50% of patients with MCA territory stroke. The reported prevalence of hyperdense artery signs is consistent with previous reports [3], [4]. We classified hyperdense artery signs as either p-HMCAS or d-HMCAS according to the site of occlusion. A proximal hyperdense artery sign was seen in 17.5% of patients. The reported incidence of d-HMCAS was 13.3% in our case series, which is similar to the findings described by Barber and colleagues [12].

The rate and extent of recanalization after intravenous rtPA thromolysis was dependent on the site and amount of thrombus. Recent study suggested that thrombus volume were significantly larger in patients with hyperdense artery signs than in those without the sign [13]. Furthermore, the authors also reported the mean thrombus volume was significantly larger in the p-HMCAS than those with d-HMCAS. Our results suggested that patients with hyperdense artery signs are less likely to have rapid neurological recovery than those without the sign. This could be well explained by the larger thrombus volume associated with the hyperdense artery signs. In our study, we compared the early neurological improvement after rt-PA thrombolysis in patients with p-HMCAS and d-HMCAS. Patient age, gender, vascular risk factors and time to treatment did not differ between patients with p-HMCAS and d-HMCAS. In addition, we also observed that patients with d-HMCAS were more likely to have early neurological improvement (median NIHSS improvement 3.5 point) than those with p-HMCAS signs (median NIHSS improvement 0 point). The results of our study suggested that intravenous rtPA is less effective in recanalizing patients with p-HMCAS.

The efficacy of rtPA in patients with hyperdense artery signs has been debated in previous reports [14]. Few studies have investigated the association of rtPA in patients with hyperdense artery signs. In a secondary analysis of 620 patients in the European Cooperative Acute Stroke Study I, Manelfe and colleagues found that patients with HMCAS who received rtPA had better neurological recovery than those who received placebo [10]. A limitation of the study is that the authors did not differentiate between proximal and distal hyperdense artery signs. The results of our study suggested that the response to rtPA was different between patients with proximal and distal hyperdense artery signs. More recently, Barber and colleagues described the efficacy of rtPA in patients with proximal and distal hyperdense artery signs. They found that 5/5 (100%) of patients with proximal HMCAS and 64% of distal HMCAS patients were either dead or dependent at 3 months [12]. However, the number is too small to draw any conclusion. The results of our study support the concept that MCA stem occlusions are less likely to benefit from rtPA treatment than MCA branch occlusions.

Differentiation between p-HMCAS and d-HMCAS is important because they have different prognostic implications. In a study of 186 patients with MCA territory stroke, Somford and colleagues reported that patients with p-HMCAS have a worse short-term outcome than those with d-HMCAS [11]. A limitation of their study is that all patients did not receive rtPA treatment in their cohort. In our study, we compared the efficacy of intravenous rtPA between p-HMCAS patients and d-HMCAS patients. We found a strong association between the presence of p-HMCAS and poor early neurological recovery after intravenous rtPA. Interestingly, we failed to find any association between d-HMCAS and poor early neurological recovery.

Previous angiographic studies suggested that disappearance of hyperdense artey signs after rtPA treatment indicates recanalization of occluded vessels [15], [16]. Disappearance of hyperdense artery signs has been associated with good functional outcome in patients who underwent thrombolysis. In a recent study, Kharitonova and colleagues reported that hyperdense artery sign disappeared in 48% of patients treated with rtPA [17]. However, the authors did not differentiate between p-HMCAS and d-HMCAS in their study. In our study, we observed that the hyperdense artery sign disappeared in 51.3% of patients treated with rtPA, which is similar to Kharitonova's finding. Furthermore, we found that disappearance of hyperdense artery sign is more common in patients with distal hyperdense artery sign than those with proximal hyperdense sign. One possible explanation is that patients with p-HMCAS may have longer clots or tandem internal carotid occlusion. Regular dose intravenous rtPA is difficult to fully recanalize those patients with a heavy clot burden [18]–[20].

The d-HMCAS sign has been verified in angiographic studies with high specificity [21]. It is well established as a reliable early CT marker of acute ischemia. However, the prognostic significance of d-HMCAS remains unclear. In previous reports, the d-HMCAS patients were pooled with p-HMCAS patients to analyse the effect of rtPA in MCA territory stroke patients. In our study, we observed that the initial neurological deficit and early neurological improvement were similar in patients with d-HMCAS and those without any hyperdense sign. Our results suggest that intravenous rtPA is equally effective in patients with d-HMCAS and those without any hyperdense artery sign. Based on our findings, we suggest that patients with hyperdense artery signs should not be investigated as a whole in future studies.

Recently, more aggressive treatment such as intra-arterial or interventional clot retrieval has been available in clinical practice [22]. The clot length is associated with the rates of intravenous tissue-type plasminogen activator recanalization. In a recent study, Kamalian et al. found that patients with internal carotid artery-terminus occlusion are likely to have clot length ≥8 mm [23]. In an observational study, Mattle and colleagues found that intraarterial thrombolysis was more beneficial than intravenous thrombolysis in stroke patients presenting with HMCAS on CT [24]. Patients with hyperdense artery signs have a heavy clot burden and should be treated with more aggressive procedures [25], [26].

Our study has several limitations. First, the data of recanalization was not assessed based on CT angiographic findings. Second, this is a single center study and the sample size is relatively small. Third, the carotid clot burden was not investigated in our present study.

In conclusion, patients with hyperdense artery signs on admission CT are less likely to have rapid neurological recovery than those without the sign. Patients with p-HMCAS are associated with more severe neurological deficit and less rapid neurological recovery than patients with d-HMCAS. P-HMCAS and d-HMCAS have different prognostic implications in patients treated with rtPA.
